# Spiritual Intelligence in Healthcare Practice and Servant Leadership as Predictors of Work Life Quality in Peruvian Nurses

**DOI:** 10.3390/nursrep15070249

**Published:** 2025-07-08

**Authors:** Paula K. Dávila-Valencia, Belvi J. Gala-Espinoza, Wilter C. Morales-García

**Affiliations:** 1 Unidad de Salud, Escuela de Posgrado, Universidad Peruana Unión, Lima 15464, Peru; 2 Dirección de Investigación e Innovación, Universidad Peruana Unión, Lima 15464, Peru

**Keywords:** spiritual intelligence, servant leadership, work life quality, nursing, structural equations

## Abstract

**Introduction:** Work life quality (WLQ) in nursing is a critical factor that influences both staff well-being and the quality of care provided to patients. Spiritual intelligence (SI) and servant leadership (SL) have been identified as potential positive predictors of WLQ, as they facilitate resilience, job satisfaction, and stress management in highly demanding hospital environments. However, the specific relationship between these constructs in the Peruvian nursing context has not yet been thoroughly explored. **Objective:** We aimed to examine the impact of spiritual intelligence and servant leadership on the work life quality of Peruvian nurses, assessing their predictive role through a structural equation modeling approach. **Methods:** A cross-sectional and explanatory study was conducted with a sample of 134 Peruvian nurses (M = 36.29 years, SD = 7.3). Validated Spanish-language instruments were used to measure SI, SL, and WLQ. Structural equation modeling (SEM) with a robust maximum likelihood estimator (MLR) was employed to evaluate the relationships between the variables. **Results:** Spiritual intelligence showed a positive correlation with WLQ (r = 0.40, *p* < 0.01) and with servant leadership (r = 0.44, *p* < 0.01). Likewise, servant leadership had a significant relationship with WLQ (r = 0.53, *p* < 0.01). The structural model demonstrated a good fit (χ^2^ = 1314.240, df = 970, CFI = 0.96, TLI = 0.96, RMSEA = 0.05, SRMR = 0.08). The hypothesis that SI positively predicts WLQ was confirmed (β = 0.41, *p* < 0.001), as was the significant effect of SL on WLQ (β = 0.26, *p* < 0.001). **Conclusions:** The results indicate that both spiritual intelligence and servant leadership are key predictors of work life quality in Peruvian nurses. SI contributes to developing a transcendent perspective on work and greater resilience, while SL fosters a positive and motivating organizational environment. It is recommended to implement training programs and leadership strategies focused on these constructs to enhance work life quality in the healthcare sector.

## 1. Introduction

Nursing is a highly demanding profession, characterized by long working hours, exposure to emotionally challenging situations, and the need to make decisions in high-pressure environments [1] . These conditions can lead to high levels of stress, emotional exhaustion, and a decline in nurses’ work life quality, affecting not only their well-being but also the quality of care provided to patients [2,3,4,5]. In this context, spiritual intelligence and servant leadership emerge as key factors in strengthening resilience, fostering a sense of purpose, and enhancing job satisfaction among nursing staff [5,6,7]. Spiritual intelligence (SI) has been defined as the ability to find meaning in adversity, transcend the immediate, and develop an integrative vision of life and work [8]. In nursing, this intelligence enables professionals to cope with stressful situations with greater resilience and empathy, fostering a sense of vocation in their daily work [9]. Recent studies have shown that nurses with high levels of SI experience greater job satisfaction, lower emotional exhaustion, and a higher capacity to manage ethical dilemmas in their work environment [10]. Additionally, SI is associated with better stress management and a more positive perception of well-being in demanding work settings [11,12].

On the other hand, servant leadership is a management model centered on the well-being and development of employees, prioritizing their needs and fostering a positive work environment [13]. In nursing, this approach has been shown to reduce staff turnover, increase job satisfaction, and improve team dynamics [14]. When servant leadership is combined with high levels of SI, values such as compassion, ethics, and commitment to patient care are reinforced, promoting a more harmonious and motivating work environment [15].

Work life quality (WLQ) in nursing refers to the perception of well-being in the workplace, encompassing factors such as workload, organizational support, work–life balance, and career development opportunities [3]. Poor WLQ is linked to high levels of stress and demotivation, which negatively affect professional performance and patient safety [4]. Several studies have explored the inter-relationship between these three concepts, highlighting that SI not only influences nurses’ QWL but also facilitates the development of effective servant leadership [14]. Specifically, research has shown that nurses with high levels of SI tend to adopt a service-oriented leadership style, as their connection to transcendent values motivates them to act with empathy and social responsibility [16]. Moreover, well-implemented servant leadership has been associated with greater job satisfaction and lower stress among nursing teams, positively impacting the QWL of healthcare personnel [17].

In this regard, the combination of servant leadership and high levels of spiritual intelligence has been linked to enhanced quality of work life, reduced stress, and greater professional satisfaction among nurses [12]. Similarly, studies conducted during the COVID-19 pandemic revealed that SI was positively correlated with well-being and life satisfaction among nursing staff, underscoring its crucial role in strengthening professional resilience [10].

Nevertheless, despite the growing body of evidence on the benefits of SI and servant leadership in demanding work environments, significant gaps remain regarding how these constructs interact in the specific context of Peruvian nursing—a setting marked by structural limitations, high patient loads, and persistent emotional exhaustion. Understanding these relationships is key not only to designing interventions that promote staff well-being but also to fostering ethical, empathetic, and sustainable leadership styles within the healthcare system. In this context, the present study aimed to examine spiritual intelligence in health practice and servant leadership as predictors of the quality of work life among Peruvian nurses. Based on this aim, the following hypotheses were proposed: (1) spiritual intelligence significantly predicts quality of work life among Peruvian nurses, and (2) servant leadership also significantly predicts quality of work life in this population.

## 2. Methods

### 2.1. Study Design and Population

This study employed a quantitative, cross-sectional, and explanatory design, aimed at analyzing causal relationships between latent variables through structural equation modeling (SEM) [18]. The target population consisted of actively practicing nurses working in healthcare centers across Peru during 2024. A non-probability convenience sampling method was used, given the limited and voluntary access to the target population. This type of sampling is common and accepted in SEM-based research, as it prioritizes adequate sample size for parameter estimation over population representativeness [19]. The inclusion criteria were as follows: (a) holding a nursing degree or equivalent qualification, (b) being actively employed at the time of the study, and (c) providing informed consent for voluntary participation. As an exclusion criterion, incomplete or duplicate responses were removed. To estimate the minimum required sample size, the following parameters were considered: a moderate number of observed and latent variables, an anticipated effect size (λ = 0.10), a statistical significance level (α = 0.05), and a statistical power of (1 − β = 0.80). Based on these parameters, the recommended minimum sample size was calculated to be 119 participants [20]. Ultimately, a total of 134 nurses participated in this study.

### 2.2. Instruments

*Work Life Quality*: The Spanish short version of the Work Life Quality Questionnaire (CVT-Gohisalo) was used [21]. The scale consists of 31 items distributed across seven dimensions: institutional support for work (6 items), job security (5 items), job integration (3 items), job satisfaction (6 items), well-being achieved through work (6 items), personal development at work (3 items), and free time management (2 items). A 5-point Likert-type response scale was used. The overall reliability of the scale showed a Cronbach alpha of 0.911, with specific values by dimension: institutional support for work (α = 0.8), job security (α = 0.5), job integration (α = 0.6), job satisfaction (α = 0.8), well-being achieved through work (α = 0.8), personal development at work (α = 0.9), and free time management (α = 0.8). In this study, the scale demonstrated an adequate fit to the data (χ^2^ = 804.46, df = 356, *p* < 0.001, CFI = 0.94, TLI = 0.93, RMSEA = 0.08, SRMR = 0.08). The reliability of the dimensions, evaluated using Cronbach’s alpha, yielded the following values: institutional support for work (α = 0.71), job security (α = 0.85), job integration (α = 0.70), job satisfaction (α = 0.85), well-being achieved through work (α = 0.81), personal development at work (α = 0.82), and free time management (α = 0.70).

*Servant Leadership*: The Spanish version [22] of the Servant Leadership Instrument, developed by Dennis and Winston [23], was used to measure servant leadership. The scale is unidimensional and employs a 5-point Likert-type measurement format. In this study, reliability was excellent, with a Cronbach alpha of 0.91 and an Omega coefficient of 0.91. Additionally, the model demonstrated an adequate fit to the theoretical framework, with the following goodness-of-fit indices: χ^2^ = 57.04, df = 44, *p* = 0.09, CFI = 0.97, TLI = 0.97, RMSEA = 0.05, and SRMR = 0.05. This confirmed a reliable measurement of the construct (α = 0.91).

*Spiritual Intelligence in Healthcare Practice*: The Spiritual Intelligence in Healthcare Practice Scale (EIEps) was used [24]. This scale consists of 18 items distributed across three dimensions: Spiritual Experience in Practice, Existential Thinking, and Transcendental Awareness. Instrument reliability was assessed using Cronbach’s alpha, obtaining values of 0.90 for the total scale, 0.84 for Spiritual Experience in Practice, 0.76 for Existential Thinking, and 0.72 for Transcendental Awareness. The scale employs a 4-point Likert-type response format. Additionally, in this study, the model demonstrated an acceptable fit, with the following values: χ^2^ = 57.760, df = 41, *p* = 0.043, CFI = 0.97, TLI = 0.96, RMSEA = 0.06, and SRMR = 0.05. The specific reliability for each dimension was also evaluated using Cronbach’s alpha, obtaining 0.82 for Spiritual Experience in Practice (SEP), 0.71 for Existential Thinking (ET), and 0.71 for Transcendental Awareness (TC).

### 2.3. Procedure

Access to the sample was obtained through coordination with healthcare institutions in Metropolitan Lima and selected regions. The nursing departments of these institutions were contacted to secure institutional authorization. Subsequently, a formal invitation was sent to potential participants via email and internal communication networks, along with a link to the questionnaire hosted on Google Forms. The first page of the instrument included a digital informed consent form. Data collection was carried out over a four-week period. All responses were anonymous and securely stored in a restricted-access environment, accessible only to the research team.

### 2.4. Ethical Considerations

This study was approved by the Ethics Committee of Universidad Peruana Unión (Approval Report: 2024-CE-EPG-00063). Confidentiality, anonymity, and voluntary participation were ensured, in accordance with the principles of the Declaration of Helsinki and the Peruvian Personal Data Protection Law (Law No. 29733).

### 2.5. Data Analysis

Descriptive statistics, including frequencies, means, standard deviations, and bivariate correlations among model variables, were calculated. The measurement model was evaluated by estimating Cronbach’s alpha and McDonald’s omega coefficients for all scales and subscales, as well as by verifying factorial validity through confirmatory factor analysis (CFA). The CFA was conducted using the robust maximum likelihood estimator (MLR) [25] due to significant deviations from multivariate normality detected using Mardia’s test. No missing data were found in the dataset (0%). Collinearity among the model’s predictor variables was assessed using the Variance Inflation Factor (VIF), with all values falling below the critical threshold of 5, indicating no significant multicollinearity [19]. Subsequently, a structural equation model (SEM) with three latent variables was specified [26], incorporating direct relationships as proposed in the study hypotheses. Model fit was evaluated using the Comparative Fit Index (CFI) and the Tucker–Lewis Index (TLI), with values > 0.90 considered acceptable [27], along with the Root Mean Square Error of Approximation (RMSEA < 0.08) [28] and the Standardized Root Mean Square Residual (SRMR < 0.08) [29].

All statistical analyses were conducted in RStudio [30] using R version 4.1.1 (R Foundation for Statistical Computing, Vienna, Austria). Confirmatory and structural analyses were performed using the “lavaan” package [31].

## 3. Results

### 3.1. Sociodemographic Characteristics of the Sample

A total of 134 Peruvian nurses participated in this study, with their ages ranging from 21 to 63 years old (M = 36.29, SD = 7.3). The majority reported having more than 10 years of professional experience (38.1%), were single (55.2%), had no children (50.7%), and held a specialist-level academic degree (50.7%). A detailed breakdown is presented in Table 1.

### 3.2. Preliminary Analysis of the Variables

Table 2 presents the descriptive statistics and bivariate correlations among the main variables of this study. All three variables demonstrated adequate internal consistency, with Cronbach’s alpha (α) and McDonald’s omega (ω) coefficients exceeding 0.90. The distributions showed negative skewness, particularly for spiritual intelligence (skewness = −2.14), which justified the use of the robust maximum likelihood estimator (MLR). Significant positive correlations were observed between spiritual intelligence and servant leadership (r = 0.44, *p* < 0.01), between spiritual intelligence and quality of work life (r = 0.40, *p* < 0.01), and between servant leadership and quality of work life (r = 0.53, *p* < 0.01).

### 3.3. Assessment of Preliminary Assumptions

Prior to conducting structural equation modeling, key statistical assumptions were assessed. First, multivariate normality was examined using Mardia’s test (skewness = 56,480.39, *p* < 0.001; kurtosis = 23.90, *p* < 0.001), with results indicating a significant deviation from normality. Consequently, the robust maximum likelihood estimator (MLR) was employed, as it is appropriate for non-normally distributed data. Additionally, no missing data were found across the observed variables (0%), confirming the integrity of the dataset. Collinearity among the structural model’s predictor variables was also assessed using the Variance Inflation Factor (VIF), yielding values of 1.28 for both spiritual intelligence and servant leadership. These results indicate no significant multicollinearity (VIF < 5), thus supporting the stability and reliability of the structural parameter estimates [19].

### 3.4. Structural Model

**Figure 1:**
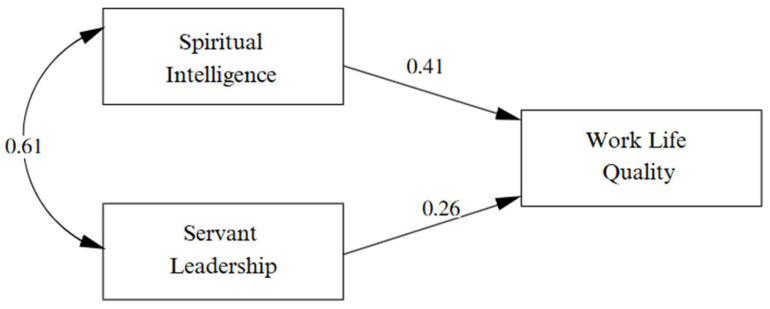
Structural model results.

The theoretical model analysis demonstrated an adequate fit with the following indicators: χ^2^ = 1314.240, df = 970, *p* = 0.000, CFI = 0.96, TLI = 0.96, RMSEA = 0.05 (90% CI: 0.04–0.06), and SRMR = 0.08 (Figure 1). The results confirmed Hypothesis 1, indicating that spiritual intelligence has a significant positive effect on work life quality (β = 0.41, *p* < 0.001). Similarly, Hypothesis 2 was supported, showing that servant leadership has a significant positive effect on work life quality (β = 0.26, *p* < 0.001).

## 4. Discussion

Nursing is a demanding profession that significantly impacts work life quality (WLQ) [4]. Spiritual intelligence (SI) and servant leadership (SL) are key factors in strengthening resilience and job satisfaction [7]. SI helps to manage stress with empathy and a sense of vocation, reducing emotional exhaustion [8,9]. Servant leadership enhances the work environment and the quality of care provided [13]. Together, they promote well-being and improve WLQ in nursing [16]. Thus, the objective of this research was to analyze the influence of SI and servant leadership on the WLQ of Peruvian nurses.

The present study confirmed Hypothesis 1, demonstrating that SI positively predicts QWL among Peruvian nurses. This finding not only supports previous research [9,11] but also contributes empirical evidence within a specific cultural context (Peru) where spiritual factors often play a significant role in shaping perceptions of work and life. Beyond corroborating earlier findings, the results emphasize that SI functions not only as a psychological protective resource against occupational stress but also as a structural factor promoting organizational well-being. In highly emotionally demanding professions such as nursing, the transcendent components of SI, such as a sense of purpose, compassion, and self-transcendence, appear to be key contributors to the perception of satisfaction with the work environment. A major contribution of this study is the validation of SI as a significant predictive variable, even after controlling for other organizational factors, suggesting that its impact is neither incidental nor solely dependent on the institutional climate. This advances prior understanding, which often treated spirituality as a personal trait with indirect effects, and instead positions SI as a professional competency that can be developed, with practical applications in staff training, recruitment, and emotional support programs in healthcare settings. Nevertheless, it is important to acknowledge that although our findings align with research linking SI to lower emotional exhaustion and greater job satisfaction [12], this study relied on self-reported measures, which may be susceptible to social desirability bias. Furthermore, while a strong relationship was found between SI and QWL, the model did not incorporate contextual variables that could add nuance this relationship, such as organizational culture, objective workload, or the type of healthcare institution. Despite these limitations, the findings suggest several practical implications. Implementing spiritual intelligence development programs in hospital settings may enhance nurses’ resilience, foster value-based leadership styles [15], and improve workplace climate. This line of intervention, aligned with servant leadership models and holistic well-being frameworks [17], may offer an innovative approach to mitigating professional burnout in high-demand healthcare environments.

Hypothesis 2 was also confirmed, revealing that servant leadership is a positive predictor of quality of work life among Peruvian nurses. This finding suggests that beyond its previously reported effects on job satisfaction [4,14], servant leadership has a broader impact that encompasses holistic workplace well-being. In highly demanding clinical environments, this leadership style may serve as an emotional and organizational protective factor. Our study provides novel empirical evidence by identifying a direct relationship between servant leadership and perceived QWL, implying that the influence of this leadership style extends beyond motivational or attitudinal aspects to the broader psychosocial dimensions of the work environment. This connection suggests that when leaders promote empathy, team development, and a shared sense of purpose, they help to create work settings perceived as safer, more meaningful, and collaborative. Moreover, the results reinforce the notion that servant leadership can buffer the negative effects of occupational stress by fostering environments where professionals feel free to express emotions, access institutional support, and participate in decision-making without fear of retaliation [11,15]. This aligns with contemporary models of transformational leadership with a prosocial orientation while going further by placing employee well-being as an end in itself—not merely as a means to achieve productivity.

### 4.1. Implications

The findings of this study offer concrete applications for improving the QWL among nursing staff. First, it is recommended that institutions implement SI development programs, featuring practical activities such as mindfulness training, workshops on meaning in work, gratitude exercises, and compassion practices. These interventions can be integrated into wellness days or occupational mental health programs and are aimed at enhancing emotional resilience, stress management, and satisfaction with the work environment. In addition, promoting a servant leadership style represents an effective organizational strategy. Training for clinical team leaders should incorporate competencies such as active listening, values-based decision-making, and empathetic emotional management. These skills not only improve the work climate but also strengthen team cohesion, reduce burnout, and foster more humanized patient care.

From an organizational management perspective, the results support the need for institutional policies that prioritize the holistic well-being of healthcare personnel. Healthcare institutions are encouraged to incorporate ongoing training into servant leadership and spiritual intelligence into professional development programs. Hospital administrators could also implement incentive systems to promote collaborative environments, reinforce a culture of mutual care, and publicly recognize leaders who contribute to team well-being.

This study adds empirical support to the growing theoretical body that positions spiritual intelligence and servant leadership as key psychosocial resources in emotionally demanding contexts. Theoretically, the findings reinforce the integration of organizational spirituality as a component of workplace well-being and suggest that its interaction with values-driven leadership styles represents a fertile area for future research. It is recommended that this relationship be explored across different healthcare settings (e.g., public vs. private, urban vs. rural) and that potential moderating variables such as professional experience, unit type, or perceived workload be taken into account. Moreover, the use of longitudinal designs would allow for the assessment of long-term effects of interventions focused on spiritual intelligence and servant leadership. A mixed-methods approach, combining quantitative techniques with interviews or focus groups, could also enrich understanding of how these factors relate to organizational culture in healthcare settings.

### 4.2. Limitations

This study presents several methodological and conceptual limitations that should be considered when interpreting the results. First, the cross-sectional design does not allow for the establishment of causal relationships. Although the findings suggest positive effects of SI and SL on QWL, only a longitudinal design could confirm directionality and detect potential changes over time. Second, the use of convenience sampling limits the representativeness of the sample. Future research is encouraged to employ stratified probability sampling and to recruit nurses from various regions, healthcare institutions, and levels of care to enhance generalizability. Third, social desirability bias may have influenced the responses, as all variables were assessed using self-report measures. To reduce this bias, future studies should consider mixed-methods designs that combine surveys with in-depth interviews, direct observation, or analysis of institutional well-being indicators. Fourth, the model did not include potential moderating variables that might influence the relationships under analysis. Future research should explore the role of factors such as perceived workload, type of clinical unit (e.g., emergency, intensive care, inpatient), professional experience, and organizational support as moderators or mediators, using structural equation models with conditional effects. Finally, the assessment of QWL relied exclusively on subjective measures. Including objective indicators such as staff turnover, absenteeism, or occupational health records would enhance the validity and practical relevance of the findings.

## 5. Conclusions

The findings of this study confirm that spiritual intelligence (SI) and servant leadership (SL) are significant predictors of quality of work life among Peruvian nurses, reinforcing their role as key resources for well-being in high-demand hospital settings. SI contributes to the development of resilience, emotional regulation, and a sense of purpose, while SL fosters a positive organizational climate characterized by empathy, mutual support, and team cohesion. Beyond their theoretical value, these results offer direct practical implications. Healthcare institutions could benefit from integrating targeted training programs in spiritual intelligence and servant leadership, aimed at both frontline staff and middle management. Such programs may include mindfulness training, care ethics, purpose-driven decision-making, and empathetic leadership skills. Investing in the development of these resources not only enhances staff well-being but is also associated with greater talent retention, reduced emotional exhaustion, and more humanized patient care. In summary, promoting SI and SL should not be viewed merely as strategies for personal growth but as essential organizational interventions to improve performance, well-being, and the sustainability of the healthcare workforce.

## Figures and Tables

**Table 1:** Sociodemographic Characteristics.

Characteristic	Category	n	%
Years of Experience	>10 years	51	38.10%
	1 year	7	5.20%
	2–5 years	32	23.90%
	6–10 years	44	32.80%
Marital Status	Married	53	39.60%
	Divorced	7	5.20%
	Single	74	55.20%
Has Children	No	68	50.70%
	Yes	66	49.30%
Educational Level	Specialist	68	50.70%
	Bachelor’s Degree	43	32.10%
	Master’s Degree	7	5.20%
	Other	16	11.90%

**Table 2:** The sociodemographic characteristics of the participants (N = 134).

Variable	M	DE	α	ω	A	K	1	2	3
1. Spiritual Intelligence	40.38	4.41	0.91	0.91	−2.14	6.55	—		
2. Servant Leadership	65.93	8.32	0.95	0.93	−1.41	2.73	0.44 **	—	
3. Work Life Quality	76.27	13.92	0.93	0.92	−0.79	0.14	0.40 **	0.53 **	—

Note. M = mean; SD = standard deviation; S = skewness; K = kurtosis; *p* < 0.01 **.

## Data Availability

The data that support the findings of this study are available from the corresponding author upon reasonable request.

## Public Involvement Statement

There was no public involvement in any aspect of this research.

## Guidelines and Standards Statement

This manuscript was prepared in accordance with the STROBE (Strengthening the Reporting of Observational Studies in Epidemiology) statement for cross-sectional observational studies [32].
